# Automatic Detection of Whole Night Snoring Events Using Non-Contact Microphone

**DOI:** 10.1371/journal.pone.0084139

**Published:** 2013-12-31

**Authors:** Eliran Dafna, Ariel Tarasiuk, Yaniv Zigel

**Affiliations:** 1 Department of Biomedical Engineering, Ben-Gurion University of the Negev, Beer–Sheva, Israel; 2 Sleep-Wake Disorders Unit, Soroka University Medical Center, and Department of Physiology, Faculty of Health Sciences, Ben-Gurion University of the Negev, Israel; King Saud University, Saudi Arabia

## Abstract

**Objective:**

Although awareness of sleep disorders is increasing, limited information is available on whole night detection of snoring. Our study aimed to develop and validate a robust, high performance, and sensitive whole-night snore detector based on non-contact technology.

**Design:**

Sounds during polysomnography (PSG) were recorded using a directional condenser microphone placed 1 m above the bed. An AdaBoost classifier was trained and validated on manually labeled snoring and non-snoring acoustic events.

**Patients:**

Sixty-seven subjects (age 52.5±13.5 years, BMI 30.8±4.7 kg/m^2^, m/f 40/27) referred for PSG for obstructive sleep apnea diagnoses were prospectively and consecutively recruited. Twenty-five subjects were used for the design study; the validation study was blindly performed on the remaining forty-two subjects.

**Measurements and Results:**

To train the proposed sound detector, >76,600 acoustic episodes collected in the design study were manually classified by three scorers into snore and non-snore episodes (e.g., bedding noise, coughing, environmental). A feature selection process was applied to select the most discriminative features extracted from time and spectral domains. The average snore/non-snore detection rate (accuracy) for the design group was 98.4% based on a ten-fold cross-validation technique. When tested on the validation group, the average detection rate was 98.2% with sensitivity of 98.0% (snore as a snore) and specificity of 98.3% (noise as noise).

**Conclusions:**

Audio-based features extracted from time and spectral domains can accurately discriminate between snore and non-snore acoustic events. This audio analysis approach enables detection and analysis of snoring sounds from a full night in order to produce quantified measures for objective follow-up of patients.

## Introduction

Partial or complete collapse of the upper airway during sleep has different effects on the human body, ranging from noisy breathing (simple snoring) [1] to obstructive sleep apnea (OSA), which can lead to considerable cardiovascular morbidity [2], [3]. Snoring is the most common symptom of sleep-disordered breathing. By age 60, snoring adversely affects 60% of men and 40% of women [4]. It is caused by the vibration of soft tissue in the upper airways involving anatomical structures such as the soft palate, uvula, and pharynx [5], [6].

The most common method for evaluating snoring history uses self-report questionnaires [4], [7], [8]. The estimated prevalence of self-reported snoring in the general population extends over a wide range from 16% to 89% [9]–[13]. This prevalence depends on awareness, age, culture, and partner complaints [4], [7], [14]. Early work has shown a poor correlation between measured loudness of snoring and subjective appreciation by different observers. It was concluded that to a large extent snoring is “in the ear of the beholder” [4]. Thus, reliable snoring reporting cannot be made based solely on a patient's (or partner's) history of noisy respiration during sleep [8], [11], [15], or with sleep laboratory technician reports [4]. An additional limitation of questionnaires is that a large portion of the subjects respond that they “do not know” if they snore [10]. To overcome these limitations, some clinicians ask the patient to supply an audio recording of their snoring, for example, prior to snore reduction surgery or to avoid operating on a “snorer” when in fact the problem lies with the bed partner being disturbed by essentially normal nocturnal breathing noise [1].

One of the main goals of sleep medicine today is to improve accessibility to sleep-disordered breathing diagnosis and treatment. The gold standard for evaluating sleep-disordered breathing is the multichannel polysomnography (PSG) study [16]. PSG on snorers, with no additional complaints suggestive of sleep-disordered breathing, will be normal in up to 80% of studies [7], [15]. However, due to difficulties associated with PSG, such as its long waiting list and costs, there is an urgent need for simple and reliable technology for snore detection and analysis. Audio signal analysis of snore sounds can be deployed in different tasks, such as assessment of the outcome of surgical treatment [17], [18]. Recently several papers have proposed OSA detection systems [19] and apnea-hypopnea index (AHI) estimation based on whole-night audio recording of snoring [20], [21]. Furthermore, in order to reliably evaluate the severity and variability of an individual's snore, the recording of an entire night is required. Hence, developing an automatic snore detection method to analyze full-night recordings in a timely and accurate manner would be advantageous.

A limited number of studies have addressed this issue of automatic detection and classification of snore signals, and even less is known about snore detection using ambient (non-contact) microphone technology. Several snore/non-snore classification methods have been suggested using different techniques to analyze snore sound events. These include pitch and formants, features regarding spectrum modeling such as Mel-frequency cepstral coefficients (MFCC), linear predictive coding (LPC) [22], and standard acoustic measures such as sound intensity [13]. Most of these studies were conducted without separate groups of subjects for their design and validation studies. Duckitt et al. [23] recorded sound with an ambient microphone from 6 subjects that was segmented into snoring episodes, breathing, duvet noise, and silence periods using hidden Markov models and spectral-based features. Cavusoglu et al. [24] proposed a method for snore detection involving 15 subjects for both design and validation study using a linear regression fed by sub-band spectral energy distributions processed by principal component analysis. Karunajeewa et al. [25] proposed a method for classifying snores and breathing sounds using the mean and covariance of four features extracted from time and spectral domains. Azarbarzin et al. [26] proposed an unsupervised snore sound extractor based on a fuzzy C-means clustering algorithm and achieved higher accuracy using a tracheal microphone, due to a higher signal-to-noise ratio (SNR) [27].

The need for an agreed upon approach to extract and analyze whole-night snoring sounds is of major importance to the field of sleep-disordered breathing. Snore sounds vary significantly; in some cases the snore sound may be soft, but in others it can be very loud [1], [4], [28]–[34]. Our study aimed to develop and validate a novel robust snore detection system (algorithm) using a non-contact technology. This detector is based on signal enhancement and features extracted from different domains as they have complementary information about snore/non-snore discrimination. The snore detection algorithm is based on three major steps: 1) Signal enhancement and segmentation, 2) Feature extraction that included specially designed novel features for characterizing snore events; the final features were selected using a comprehensive feature selection technique that automatically revealed the most prominent features, and 3) Detection of snore events using an AdaBoost classifier [35] that was trained using thousands of snore and non-snore events. The novelty of our proposed method is its automatic detection of every snore event from the whole-night audio recording using non-contact technology. Moreover, this approach includes comprehensive sets of features involving time and spectral domains, which were selected using a feature selection algorithm. In addition, we propose an objective score for quantifying snore intensity.

## Methods

This article has online Methods Supporting File S1.

### Setting

A university affiliated sleep–wake disorder center and biomedical signal processing laboratory. The Institutional Review Committee of the Soroka University Medical Center approved the study: protocol number 10621. Informed consent was obtained from all subjects.

### Subjects

We prospectively recruited 67 consecutive adults (aged 19 to 87 years, 27/40 women/men) referred to the Sleep–Wake Unit at Soroka University Medical Center in Beer Sheva, Israel for routine polysomnographic (PSG) sleep-disordered breathing diagnosis, starting in February 2008. We selected the first 25 subjects (patients) for the system design (training) study. The remaining 42 subjects (beginning in May 2009) were included in the blind validation study.

### PSG study

Prior to nocturnal in-laboratory PSG, all subjects completed a validated self-administered sleep questionnaire [3], [36]–[38]. The Epworth Sleepiness Scale (ESS) was used to evaluate daytime sleepiness [39]. Overnight PSG was performed according to previously described methods [3], [40]. Subjects reported to the laboratory at 20:30 and were discharged at 06:00 the following morning. They were encouraged to maintain their usual daily routine and to avoid any caffeine and/or alcohol intake on the day of the study. Shift workers did not perform the PSG study in the week following their shift duty. The PSG study included electroencephalography, electrooculography, and electromyography applied over the submental muscles and bilateral anterior tibialis muscles for detection of periodic limb movements, electrocardiography, respiratory activity (abdomen and chest efforts belt), oxygen saturation, and snore level intensity (Quest Technology 2700, Orlando, FL, USA). PSG scoring was done by a trained technician and underwent a second scoring by one of the investigators (AT). Apneas and hypopneas were scored according to the American Academy of Sleep Medicine criteria [16].

### The experimental system

**Figure 1:**
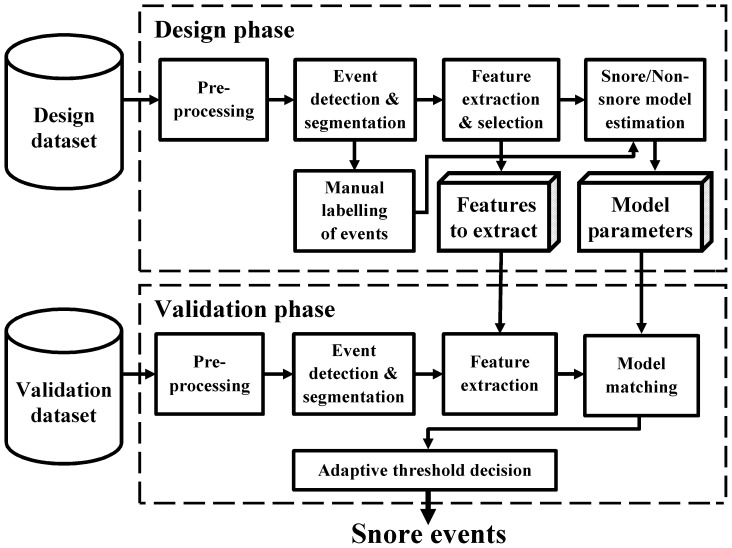
Block diagram of the study protocol. Upper panel – design phase (*n* = 25). Lower panel – validation phase (*n* = 42).

We have developed a system for snore detection that utilizes a non-contact microphone for audio signal recordings taken from a full night at a sleep laboratory. The acquired audio signals were further used to develop the snore detection algorithm. A digital audio recorder (Edirol R-4 Pro, Bellingham, WA, USA) with a directional microphone (RØDE, NTG-1, Silverwater, NSW, Australia) placed at a distance of 1 meter above head level was used for audio recording. An additional audio recorder (handy *Olympus LS-5*) was placed on the dresser beside the patient's head in the laboratory and used to validate the system in a non-laboratory setting. The audio signals were stored along with the PSG signals for later analysis. Each audio signal was synchronized with the PSG study at 15 ms resolution. The synchronization was performed via a cross-correlation technique between the PSG snore intensity level channel and the digital audio signal (after energy extraction). The main purpose was to synchronize all PSG channels to the digital audio signal, including hypnogram and respiratory effort channels, to support and assist the manual labeling of snore events. Fig. 1 shows the block diagram of the proposed snore detection algorithm for the design and validation phases of the study. In the snore detection algorithm, an adaptive noise reduction algorithm was applied for signal enhancement in a pre-processing stage. For system design, snore and non-snore events (see below) were manually labeled and used to train an AdaBoost classifier (see below) fed by acoustic features from time and spectral domains. Using a feature selection algorithm on the design group, the best features were selected and used in the validation phase as well.

### The Snore Detection Algorithm

#### Pre-processing (Fig. 1)

For design and validation phases, the acquired audio signals recorded in the sleep lab were digitized at a sampling frequency of 44.1 kHz, PCM, and 16 bits per sample, which is the minimum sampling rate of the audio recorder. All audio signals were down-sampled to 16 kHz, and each audio signal underwent an adaptive noise suppression (spectral subtraction) process based on the Wiener-filter. This process relies on automatically tracking background noise segments in order to estimate their spectra and subtracting them from the audio signal [41]. In this study, a noise spectral template was subtracted from each audio frame (40 ms). This template was initially estimated from the lowest energy frame of the first 10 sec of the audio signal and was updated during the adaptive noise suppression process. Each frame's frequency component was suppressed by a specific value (suppression factor) derived from the noise spectral template, and it was limited to the range [0, −25 dB] in order to prevent a major distortion when low SNR was present (see a detailed description in the online supporting File S1).

#### Event detection and segmentation (Fig. 1)

Audio events (snore and non-snore events) were automatically detected and segmented using an adaptive energy threshold. These audio events were segments with higher energy compared to the remaining (diminished) background noise in the audio signal. A detailed block diagram of the event detection and segmentation module is presented in the online supplement (Fig. S1 in File S1). Initially, the full-night audio signal was divided into one-minute sections, and for every section, an energy vector was calculated using energy frames (frame size: 60 ms with 75% overlap). A section-related energy threshold (adaptive threshold) *e_th_* was calculated using a histogram of the energy vector *hist_energy_*(*e*). Since the prevalence of the (remaining) background noise frames was greater relative to the audio events (snore/non-snore), the energy value related to the peak of the histogram *e_max,_* was located on the low energy scale:
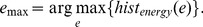
(1)The energy threshold (*e_th_*>*e_max_*) was set to the energy value corresponding to one-tenth of the peak amplitude:
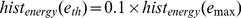
(2)


**Figure 2:**
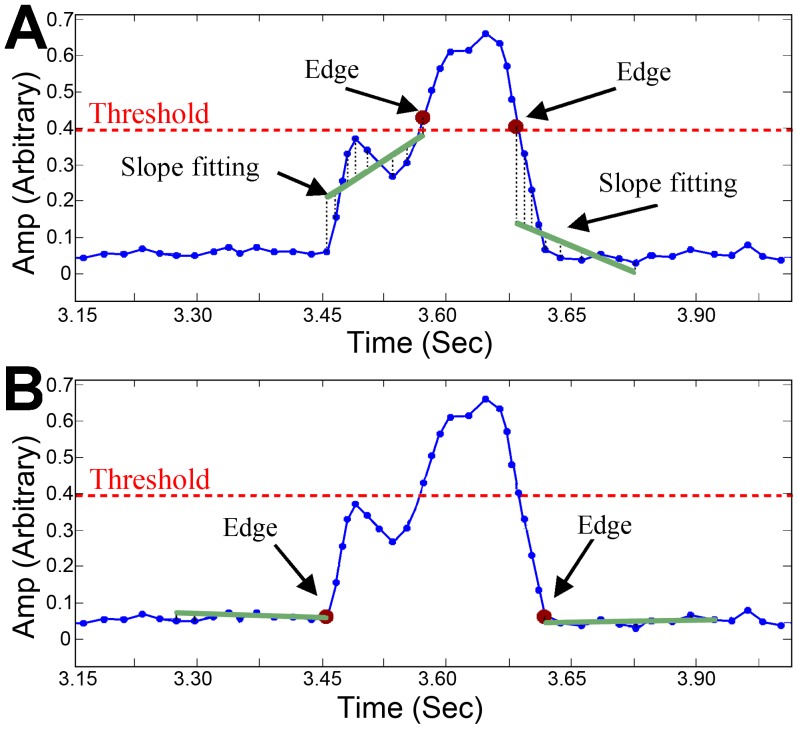
Audio event (snore/non-snore) segmentation. A) The initial segmentation edges of the audio event. B) The modified segmentation edges when applying the segmentation procedure. The green solid line represents the slope fitting of ten consecutive frames from both sides of the event. This process is repeated and progressed outside the event boundaries one frame at the time for as long as the fitted slope did not change its sign (±).

**Figure 3:**
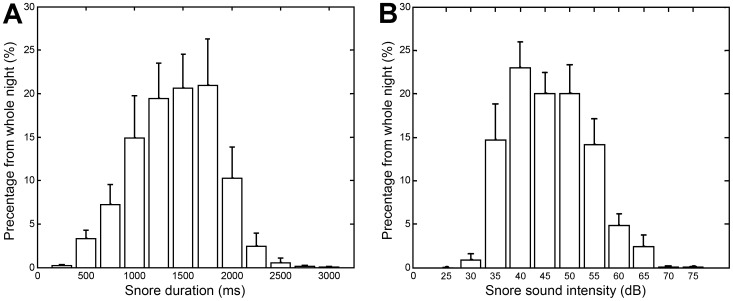
Snore characteristics. A) Snore duration, B) Snore intensity. Data was collected during design phase (*n* = 25). 99.9% of snoring event durations were in the range of 200 to 3500 ms. 99.2% of the snore intensity was in the range of 25 dB to 75 dB.

A five-order median filter was applied to the threshold values (vector) to smooth outliers. *Event detection* – Energetic audio events were detected as a group of consecutive energy frames that surpassed the section-related threshold. *Event segmentation* – In order to find the exact event boundaries (edges), the time edges of each audio event were calculated using an estimated slope technique. An illustration of the segmentation process is shown in Fig. 2. This technique included the estimation of a slope from ten consecutive energy frames (150 ms window) – a linear regression fitting line was calculated from the consecutive energy frames in order to estimate its slope. This process was repeated and progressed outside the event boundaries one frame at a time for as long as the slope did not change its sign. Next, a *fragmentation test* was applied. In case the detected audio events were too close to each other (<200 ms), they were suspected to be one fragmented event (such as split snores). This fragmentation test involved a spectral similarity measure for the 100 ms adjacent windows of the suspected events (the ending part from the first event and the initiating part from the following event). In case of similarity, the events were merged to form one event. Finally, an *event duration test* was applied – only 200 ms to 3500 ms events were used in this study since we noticed that the duration of >99% of the manually labeled events fell in this range. Fig. 3 shows snore statistics based on manual labeling of snoring events. For more information and demonstration of the event detection process see Fig. S1 and S2 in File S1.

#### Manual labeling of events (Fig. 1)

**Figure 4:**
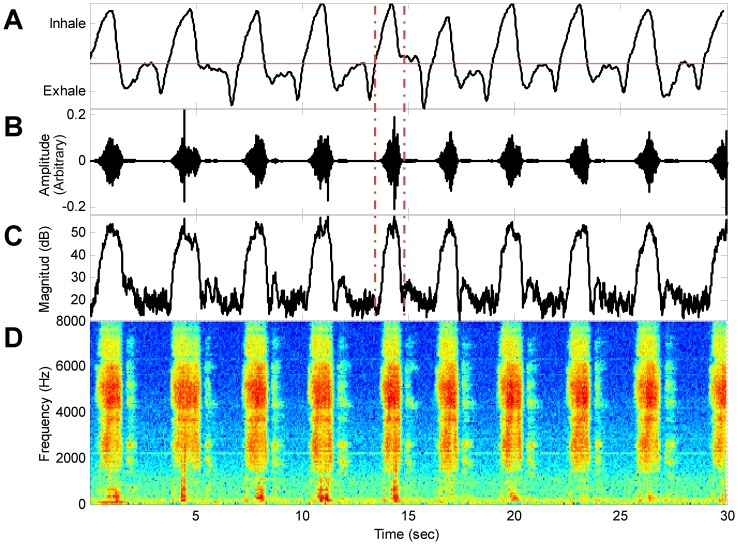
Example of snoring pattern during 30 sec epoch. A) Air flow, B) Audio signal, C) Energy signal, D) Spectrogram. Dashed vertical lines highlight one inspiratory event. Note that snore events are predominantly apparent during the inspiratory phase of the respiratory cycle. Data was collected from a 57-year-old man (BMI = 31, AHI = 16, during sleep stage 2).

This stage was essential for designing and evaluating the snore detection algorithm. Therefore, an ad hoc graphical user interface (GUI) for manual event classification based on visual and acoustic perception of the event itself and its surrounding context was designed. In this GUI, audio signals were presented in the time and frequency domains accompanying the synchronized PSG's respiratory effort signals. Using these signals, we were able to label each audio event as an inspiratory sound (snore) or non-inspiratory sound (exhale sound or noise). Fig. 4 shows an example of signals from a 57-year-old man; note that the prominent audio events (Fig. 4B) were inspiratory episodes.

Three research assistants (including one of the authors, ED) performed manual annotation of the audio signals in order to train and evaluate the snore detection system. All assistants were guided by a sleep expert (AT) in the annotation protocol in order to make sure that the definition of snore/non-snore was clear.

Two classes of audio events were defined in the annotation protocol: snoring and non-snoring. Snoring was defined as a breathing sound that occurred during an inspiration (that was visualized by the PSG's respiratory belt movements in the GUI) with an intensity >20 dB (the minimum sound detection sensitivity of our audio recording setup). Nevertheless, breathing sounds may occur during inspiratory and/or expiratory episodes. However, in the current study, the inspiratory sounds were chosen as snores since we found that 97.5±2.2% (mean ± SD) of noisy breathing cycles during sleep were composed of an inspiratory sound that was much louder than the expiratory sound. Moreover, often expiratory sounds were not observed at all (Fig. 4). Non-snore events were defined as events that did not follow the snore definition, such as bedding noise, coughing, talking, and other environmental noises.

According to the manual snore/noise annotation protocol, agreement measurements (Cohen's kappa [42]) between the three scorers were calculated. *κ_12_* = 97.4%, *κ_13_* = 97.9%, and *κ_23_* = 97.0% are the agreement scores between scorers 1 and 2, scorers 1 and 3, and scorers 2 and 3, respectively. Discrepancies were resolved by additional revision by the scorers. The majority of these proved to be human error (>98% – accidently pressing the wrong classification button), and the rest (<2%) were resolved by a majority vote of the three scorers.

#### Feature extraction (Fig. 1)

We established a pool of 127 features relevant for distinguishing snore events from non-snore events. Table 1 summarizes these feature categories based on time- and spectral-related domains using intra/inter-event properties. A detailed description of the entire set of features is available in the online supporting File S1.

**Table 1:** The entire (127) feature categories.

Feature categories	Number of features	Selected features
**I. Time-related features**	25	10
a) Periodicity features (Inter-events)	10	6
b) Duration and sample scattering (Intra-events)	4	1
c) Energy features (Intra-events)	11	3
**II. Spectral-related features**	102	24
a) Spectral parameterization (Intra-events)	68	16
b) Bio-characteristic frequencies (Intra-events)	10	4
c) Dynamic frequencies features (Intra-events)	24	4

The entire set of features is divided into time- and spectral-related domains. Note that in parentheses are the inter/intra characteristics of the feature category. A detailed description of the entire set of features is available in the online supporting File S1.

**Figure 5:**
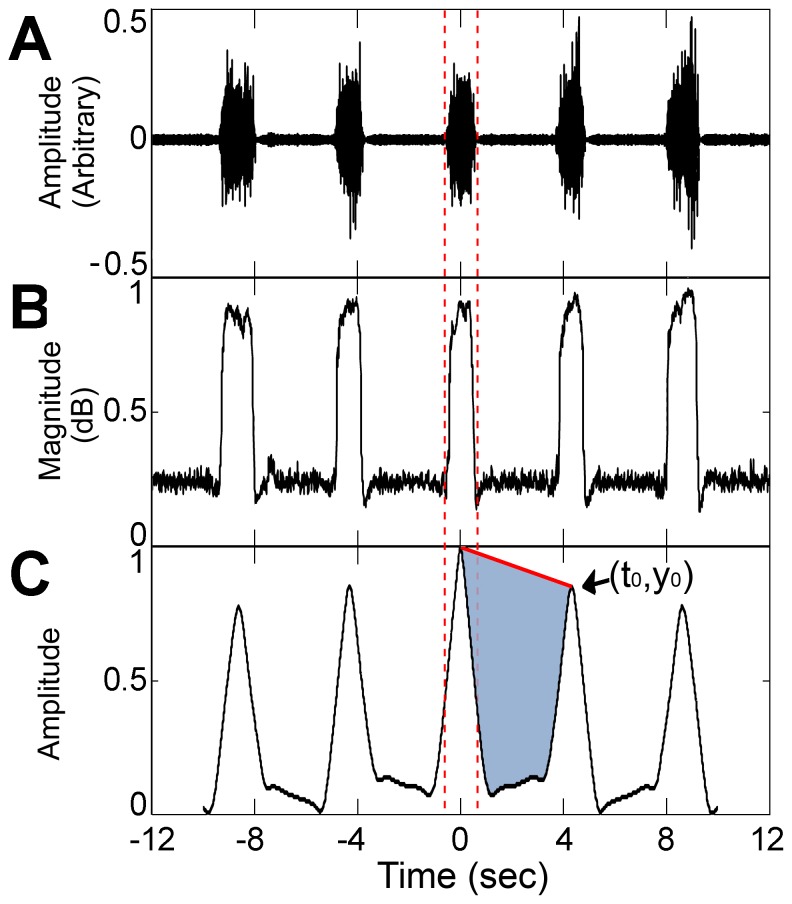
Demonstration of the major periodicity features. A) Audio signal containing snore pattern. B) The energy signal – achieved by a logistic transformation over the standard energy signal to emphasize the rhythm pattern. C) Smoothed autocorrelation R(τ) of the segment. In this example, the rhythm period was equal to t_0_ = 4.1 seconds with intensity of y_0_ = 0.8. Note that the tested event is aligned at t = 0 and t = 12 seconds for each side being tested. The shaded area in C represents the period's intensity feature. A more periodic energy pattern will result in a greater area and, hence, a greater period intensity value. The features were calculated for each tested event (between dashed vertical lines) by exploring the event's surroundings.



A brief description of the feature categories:

- ***The time-domain set –*** constructed from features that quantify parameters regarding the event's timing and their location relative to adjacent events. Twenty-five features, separated into three sub-categories, are included in this set:
  - ***The periodicity features*** seek a snoring pattern (repeated events) calculated via autocorrelation, *R*, of an energy signal interval (with a pre-defined duration), which includes the event surroundings (Fig. 5). The first peak of the estimated autocorrelation function within the range of 1 sec to 10 sec was selected because it holds information about the basic rhythm period (i.e., location of the peak τ_p_) and its power (its amplitude relative to the zero-lag autocorrelation *R_0_*). The *rhythm period* feature was calculated as τ_p_ and the *period's intensity feature R_I_* was calculated as the product of the first peak amplitude value *R*(*τ_p_*) and the initial correlation area *Area*.

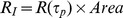
(3)where *Area* is actually the normalized square area between the *R*(*τ*) curve and the *aτ+*1 linear line from *R*(0) to *R*(*τ_p_*) calculated as:
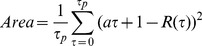
(4)where *a* is the estimated linear slope. The more periodic the energy pattern, the greater the *period's intensity feature* (Fig. 5). Another important feature is *relative energy prior to event E_P_*, which was calculated as:
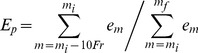
(5)where *e_m_* represents the energy signal at frame index *m*, and *m_i_* and *m_f_* represent the initial and final frame index of the tested event, respectively. *Fr* represents the frame rate (10*Fr* is the 10 sec interval prior to the tested event). This feature uses additional statistical information about snoring template/rhythm. During snoring episodes, the energy signal interval that comes prior to the tested snore event includes a certain number of snores corresponding to the breathing period; normalizing the total energy of this interval [the nominator of Eq. (5)] with the total energy of the tested event [the denominator of Eq. (5)], yields a value that is an estimation of the number of similar events in this prior interval, resulting in a relatively low value and a narrow dynamic range of *E_P_* (4.6±2.0). In noisy environments (non-snores), *E_P_* usually receives high values (27.0±20.0).

-
  - ***The duration features*** include parameters such as the *whole event*'*s duration* (in seconds) and its *95% energy duration* (in seconds). In some cases (especially in OSA snores), the difference between these two can be significant.
  - ***The energy features*** involve two major sub-sets: event-based and frame-based features. Event-based energy features are those regarding an event's total energy such as the event's intensity and SNR on the dB scale. Frame-based energy features include those that quantify and parameterize the shape and the formation of the event's energy over time.
- ***The spectral-domain set –*** consists of features extracted from the signal frequencies' components. This set includes 102 features spread among three sub-categories: parameterization of the models describing the signal spectrum, bio-characteristic frequencies regarding vocal tract parameterization (modeling), and the dynamics of the frequencies' components: a) *The Spectral parameterization* consists of several spectral models such as linear prediction coding (LPC), Mel-frequency cepstral coefficients (MFCC) [22], and sub-band frequency distribution. For each model, we also included the first four statistical moments (1-mean, 2-variance, 3-skewness, and 4-kurtosis). b) *Bio-characteristic frequencies* include features that estimate the vocal tract formation such as *formants*, *pitch*, and pitch-related features [43], [44]. c) *Dynamic frequencies* aim to measure changes in event frequency components. Usually, a snore's spectrum is more stationary in comparison to other noises (when spectral subtraction has already been performed).

#### Feature selection

A feature selection algorithm [45] was applied (Fig. 1). This was performed in order to reduce the complexity of the snore detection algorithm, to improve its performance, and to avoid over-fitting. A forward feature selection (FS) was conducted on the complete set of 127 features. The criterion that was chosen for the feature selection algorithm was the detection accuracy (snore as snore and non-snore as non-snore) that was accomplished using the AdaBoost classifier decision (see below) compared with the manual labeling of the events. We used the 10-fold cross-validation method [45] on the design dataset in order to determine the optimal number of features and the corresponding feature subset, and the resubstitution method in order to estimate the upper bound of the system's accuracy (detection rate). The optimal selected features were used in the validation phase of the study.

#### Classifier parameters estimation

An AdaBoost classifier [35] was used. Generally, a *k*-order AdaBoost classifier involves binary discrimination of *k*-weak learners, meaning *k* simple rules (thresholds) in a *d*-dimensional feature space (in our case *d* = 34) based on the true labeling of the events. Classification decision (pattern matching) was made using a decision score that is calculated via weighted summation of the *k*-weak learners. In the design phase, the classifier parameters were estimated (Fig. 1) to discriminate between two classes: snore and non-snore events. In order to estimate the classifier's parameters, the aforementioned manually labeled events were used. As mentioned above, the AdaBoost decision was also used for the feature selection criterion. For this purpose, the order was set to *k* = 100, which was found to be efficient yielding reliable results and a reasonable time-consuming feature selection process. For the purpose of snore detection (the overall system), the optimal order was found to be *k* = 300. This order was determined using the selected features (*d* = 34) yielding the best results for the design dataset and avoiding over-fitting.

#### Pattern matching (Fig.1)

The classification decision was made using a decision score, *S*, calculated via weighted summation of the *k*-weak learners. Hence, the tested events were given a detection score, measuring their similarity to the (trained) snore/non-snore properties. During the design phase, snores were assigned the value “+1” and non-snores “-1,” producing a linear estimation within that range, i.e., a snore event is likely to have a more positive score than a non-snore event. During the validation phase, a detection score was calculated for each detected audio event (for each of the 42 subjects).

#### Individual decision score threshold (Fig. 1)

**Figure 6:**
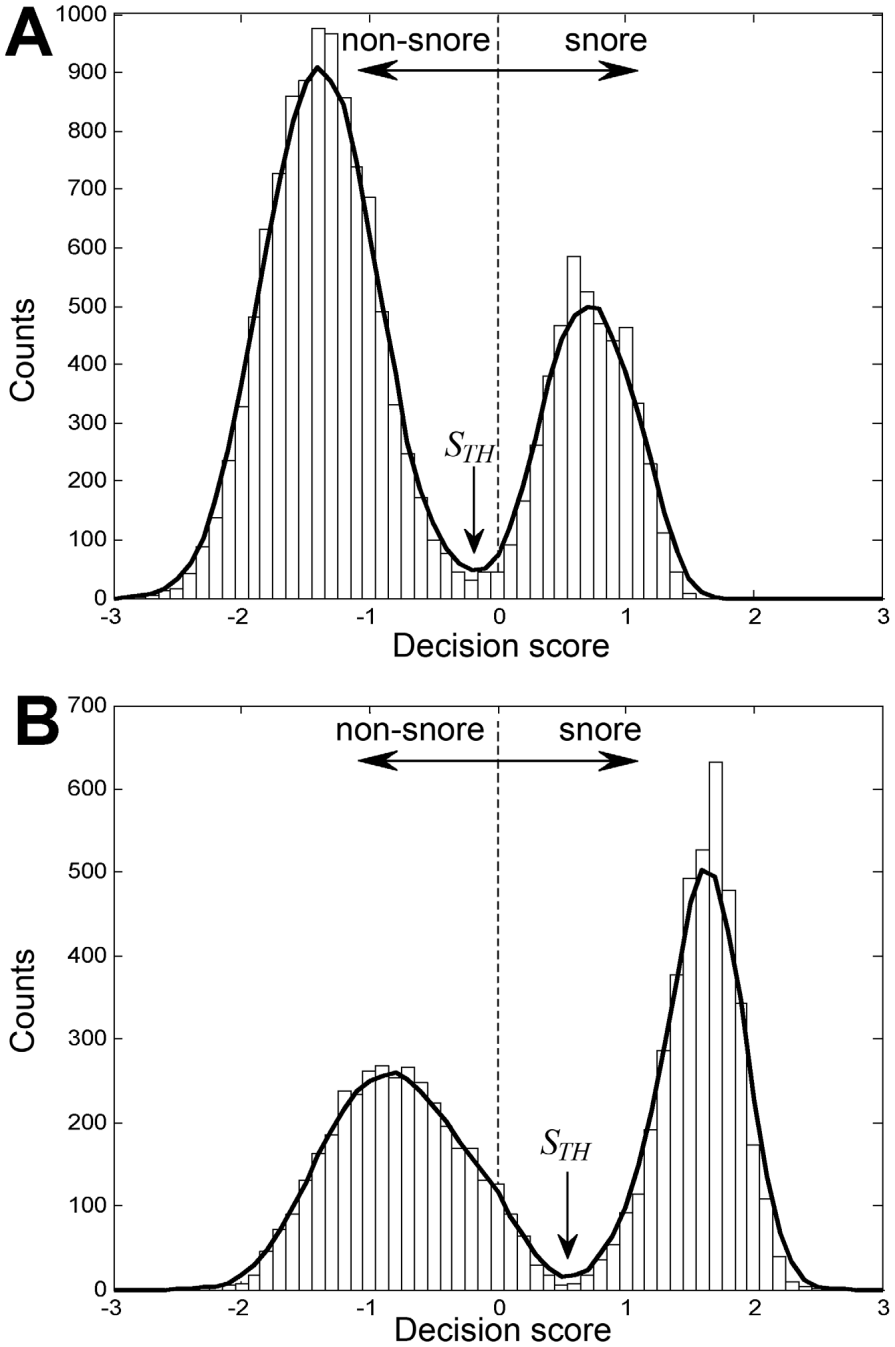
Individual decision score threshold for snore/non-snore events from two subjects. A) Example of subject with scores slightly shifted to the left (i.e., prone to noise detection). B) Example of subject with scores slightly shifted to the right (i.e., prone to snore detection). To avoid a false decision, the search of the minimum point was limited to a narrow region around zero. When only one type of event is present or no significant “valley” was found, a zero threshold was selected. Columns represent the histograms of the overall event scores. Bold lines represent a smoothed five-order moving average low-pass filter. The dashed line represents the default decision threshold, and *S_TH_*, represents the calculated score threshold.

Since snoring properties can vary between subjects, we found that applying an individual decision score threshold improved system performance. Accordingly, for each subject, a score threshold (*S_TH_*) was calculated using the scores of the subject's detected events. This score histogram was considered to behave as a bi-modal distribution since it is composed of snore and non-snore scores. The threshold was determined as the score value (index) of the minimum (“valley”) point in the histogram (Fig. 6).

### Data and Statistical Analyses

Audio signal processing and statistical analyses were performed using MATLAB (R-2010b, The MathWorks, Inc., Natick, MA, USA). Both the system design study (*n* = 25) and the validation study (*n* = 42) had similar data handling protocols. Statistical power (*α* = 0.05) was calculated for the validation set based on event score values extracted from the system design data set. A sample size of 40 subjects was calculated to provide a statistical power of 0.88 in order to achieve a system accuracy rate of >97%. Therefore, 42 subjects were recruited for the validation study. PSG, demographic, and audio data were compared between design and validation study groups using unpaired student *t*-tests or χ^2^ tests. The relationship between the subjective impression of subjects (questionnaires) and the objective measure of snoring was assessed with the help of Spearman correlations. The snore/non-snore classification performances of the design study involved resubstitution and ten-fold cross-validation methods. The snore/non-snore classification performances of the validation study involved a hold-out method, in which the system training was performed using the design dataset, and system validation was performed on the validation dataset. Detection performances were calculated using sensitivity, specificity, and accuracy:
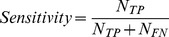
(6)where *N_TP_* represents the number of detected snores as snores (true positive), and *N_FN_* is the number of events corresponding to the false detection of snores as non-snores (false negative).
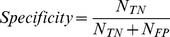
(7)where *N_TN_* represents the number of detected non-snores as non-snores (true negative), and *N_FP_* is the number of events corresponding to the false detection of non-snores as snores (false positive).
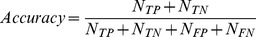
(8)


By observing the intensity of patients' snores, we arbitrarily divided the intensity into three levels: <40 dB, ≥40 dB ≤55 dB, and >55 dB, according to the distribution of the snoring intensity. This data can be presented using histogram bars and, hence, provides useful information about an individual's snore intensity. Moreover, a snore intensity score was calculated for each subject, namely *objective snore intensity* (OSI). This score was calculated as the mean of a whole-night's snore intensity (in dBs) of an individual subject based on his/her detected snores. Finally, performances for different working points were obtained from a receiver-operating curve (ROC) and the area under this curve (AUC). Data are presented as mean ± SD.

## Results

No significant differences were found between system design (*n* = 25, m/f 14/11) and validation (*n* = 42, m/f 26/16) groups in subjects' demographic characteristics, age, BMI, snoring, ESS, or AHI (Table 2). During the PSG study, an average of 7.5±1.1 hours of audio signals were recorded from each subject with no significant differences between the design and validation studies. A total of 180.3 hours and 324.2 hours were analyzed in these studies, respectively, and a total of 39,025 and 68,367 snore events were identified from the audio signals in them, respectively. Additionally, a total of 37,712 and 136,849 non-snore events were identified in each study, respectively. The non-snore events consisted of: 1) biological sources such as breathing (exhale), talking, murmurs, groaning, moaning, and coughing; and 2) other sources such as bedding noises, noise from electric devices, and slamming doors.

**Table 2:** Subject Characteristics.

	System Design (n = 25)	System Validation (n = 42)	*p*
Gender (M/F)	14/11	26/16	0.633
Age (yr)	53.1±11.7 (29–82)	52.2±14.5 (23–81)	0.793
AHI (events/hr)	18.9±18.0 (2.0–64.9)	20.3±16.2 (0.5–74.4)	0.743
BMI (kg/m^2^)	31.1±3.3 (26.7–38)	30.6±5.3 (16.8–39.3)	0.672
ESS (score)	10.3±6.4 (0–19)	13.7±6.5 (0–23)	0.573
Total labeled snoring events (×1000) (M/F)	23.0/16.0	46.3/22.0	0.366
Labeled snoring index (events/hr)	395.2±241.3	412.9±256.2	0.780
Total labeled noise events (×1000) (M/F)	23.4/14.3	87.0/50.1	0.880

AHI – apnea hypopnea index, BMI – body mass index, ESS – Epworth sleepiness scale. All values are mean ± SD (range).

### Preprocessing

Background noise removal proved to be beneficial in enhancing the signal and emphasizing faint and hidden acoustic events. On average, the enhancement of each acoustic event was improved by +6 dB, supporting earlier studies [41], [46]. While the time and spectra domains seemed to be dramatically improved in the entire audio signal, some small distortion was registered (can be detected by ear) on the lower SNR events. Nevertheless, the enhancement contribution was unquestionable throughout the entire process described in Fig. 1.

The event detection process proved to be very sensitive, followed by a high detection rate. Compared with manually marked detectable events, the positive detection rate (*PD*) was 100%, and the positive predictive value (*PPV*) of 23% was achieved – meaning false-alarm detection was four times that of true detection. Nevertheless, the high false alarm events were easily eliminated by the duration test (200 ms to 3500 ms) and the snore/non-snore classifier itself.

### Feature selection

**Figure 7:**
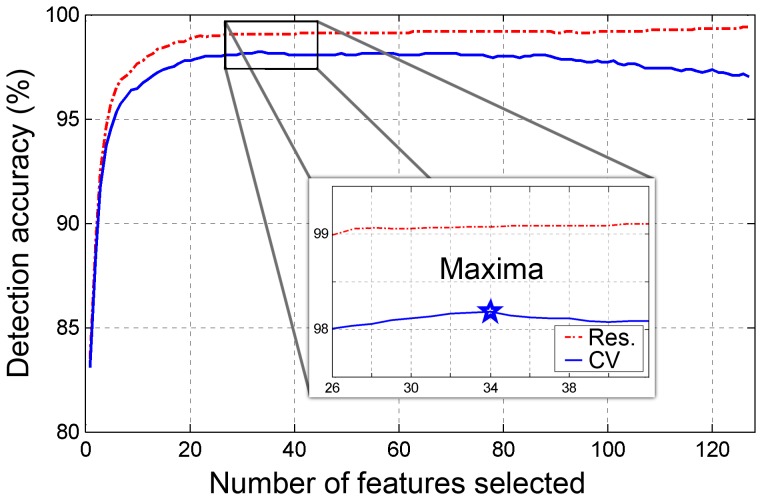
Criterion curve of the feature selection algorithm. The inner window is a zoom in at the maximal value. Thirty-four features were selected. The dashed-dotted line (red) represents the resubstitution (Res) method performances, and the solid line (blue) represents the 10-fold CV performances (CV).

We used the proposed AdaBoost-based method for the snore/non-snore detection algorithm. Fig. 7 shows the performance of the forward feature selection process conducted for the design study. The optimal performances were achieved using 34 features extracted from the complete set of 127 features (Table 1).

The 34 selected features contained representatives from two feature domains: time and frequency. The three most significant selected features (descending priority) derived from the forward feature selection process were: *(1) Relative energy prior to event (E_P_), (2) Rhythm intensity of* ±*12 seconds (R_I_), and (3) The first formant frequency.* Note that the first three significant features were extracted from the two domains explored (time and spectra). The most discriminative feature that was selected is *E_P_*. The second complementary feature was the rhythm intensity related to the ±12 sec surrounding the events. The information from this feature emphasizes the first feature in the absence of a rhythmic pattern such as snoring episodes. Even though OSA patients disrupt this rhythm during apneic events, they still have some consecutive snores prior to such an event. For detailed selected features, see online supporting information Tables S1 and S2 in File S1.

### Performance evaluation

**Figure 8:**
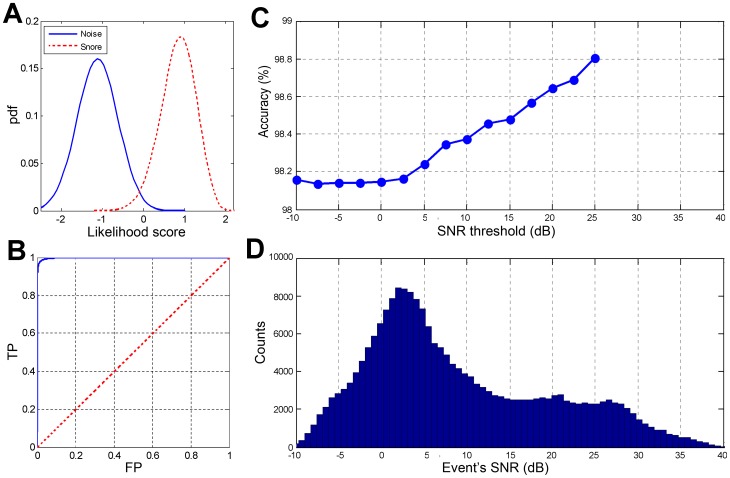
Performance of the snore detection algorithm: A) An event's likelihood score distribution (pdf – of true snores and non-snore events). B) ROC curve – detection rate True positive (TP) vs. false positive (FP) snores; area under the ROC curve = 0.998. The dashed line represents the “random guess” performance. C) The overall detection rate is based on different signal-to-noise ratio (SNR) thresholds of events. D) SNR distribution of overall events.

**Figure 9:**
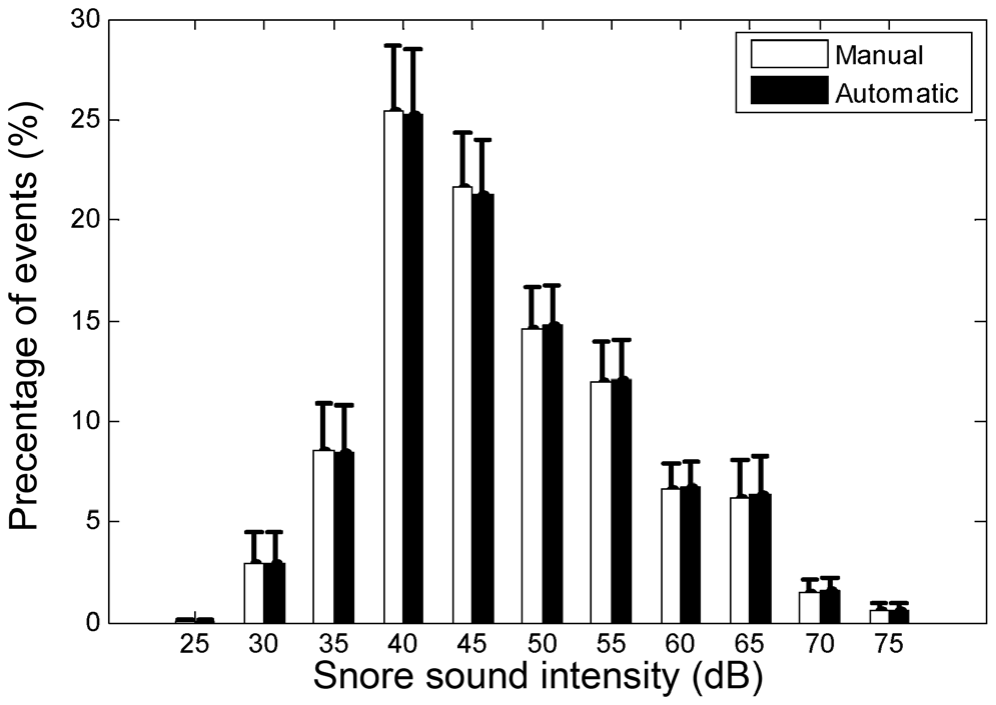
Snore intensity distribution of the validation dataset (n = 45). Open and closed bars display the distribution of snoring intensity on manually segmented events and automatically segmented events, respectively. Note that no consistent bias was found between manually and automatically segmented snore events. Error bars are standard error.

Using the chosen features, an evaluation test was conducted on the validation dataset. During the validation phase, score threshold (*S_TH_*) was calculated for each of the subjects. Each subject's scores (for snore and non-snore events) were shifted using this threshold value, allowing a global alignment of the scores from all the subjects. Hence, the evaluation of the overall performance of the system is feasible. Fig. 8A shows an event's likelihood score distribution (pdf) corresponding to their true labels (snores and non-snores). The corresponding ROC curve is presented in Fig. 8B and C. The overall detection rate (accuracy) was 98.2% with sensitivity of 98.1% (detecting snores as snores), and specificity of 98.2% (detecting noise as noise) with a confusion matrix as shown in Table 3. In addition, we measured performance as a function of the event's SNR, with the assumption that in using only relatively high SNR events (as in most previous studies), the performance of the system will be superior. In our study, the overall performance was calculated using very low SNR events as well in order to detect all snore events, even the very soft ones. The results are shown in Fig. 8C and D. Fig. 9 presents the distribution of snore intensity (5 dB resolution) for the validation dataset (manual and automatic snore detection). In this examination, we investigated the patient's snore intensity distribution throughout the night. The error bars represent the diversity of the participating subjects. By comparing the distribution of manual (open bars) vs. automatic (closed bars) detection of snore intensity, no significant bias was observed in each of the intensity sub-bands.

**Table 3:** Table 3. Classification results.

Classified as	Snore	Noise
True label		
Snore	98.1%	1.9%
Noise	1.8%	98.2%

**Figure 10:**
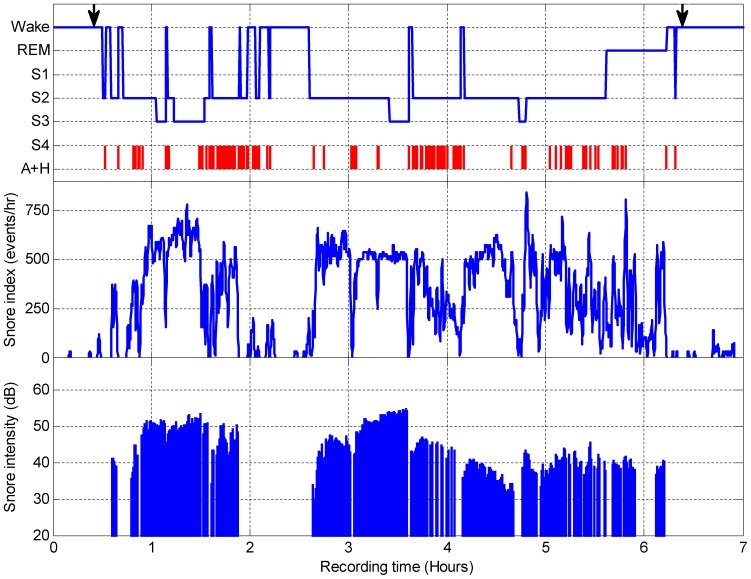
Example of whole-night snore index and snore intensity. Upper panel – sleep stages throughout the night and apnea/hypopnea events (A+H) based on the PSG test. Middle panel – automatically detected and calculated snoring index (events/hr) per 30 second epoch. Lower panel – snore intensity (dB). Arrows indicate lights off and lights on, respectively. Data was collected from a 63-year-old woman (BMI = 31, AHI = 14). For this subject only, a snoring index >240 events/hr is displayed.

Fig. 10 shows an example of full-night recording. Data was collected from a 63-year-old woman (BMI = 31, AHI = 14). Snore intensity using the dB scale was measured for each of the detected snores. The snore index was defined as the running number of detected snore events per hour, estimated from 30 sec intervals (for each epoch). Snore intensity and snore index were aligned with the hypnogram, which was calculated using PSG analysis. Note that the snore index dropped considerably during wakefulness and periods of AHI.

**Figure 11:**
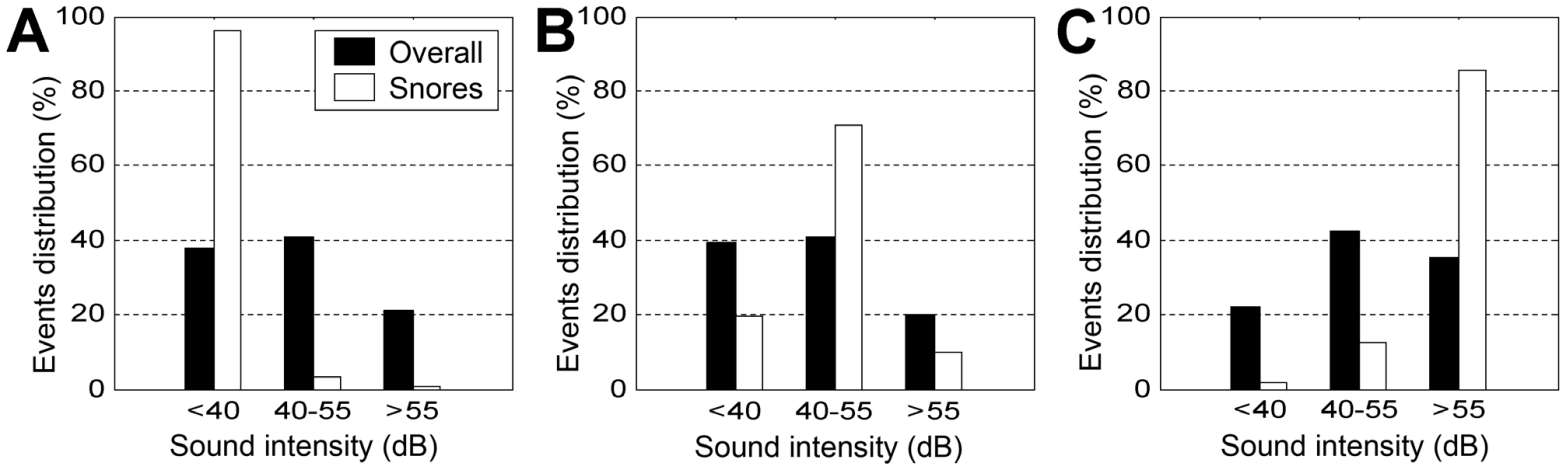
Example of sound intensity distribution for three subjects (arbitrarily selected) during the validation phase of the study. Closed bars are the sound intensity of all the audio events (snore and non-snore events) between “lights off” and “lights on.” Open bars are the sound intensity of the detected snoring events. Data are presented as a percentage. In this example, subjects A, B, and C have objective snore intensity (OSI) of 37 dB, 46 dB, and 59 dB, respectively.

Fig. 11 shows three examples of sound intensity histograms from three subjects during the validation phase of the study. The sound intensity of all audio events, i.e., snore and non-snore intensity, prior to snore detection (closed bars) had a similar distribution between subjects. Similar distribution characteristics were found for all 42 subjects included in the validation phase. However, observing the intensity of the automatically detected snoring events (open bars) reveals significant differences of snoring intensity among subjects. Fig. 11A shows a case where 92% of the snoring events of this subject were below 40 dB, suggesting a light snorer. On the other hand, Fig. 11C shows a case where >86% of the snoring events were above 55 dB, suggesting a loud snorer. Spearman correlation revealed no correlation between objective snore intensity and subjective self-reported snore intensity (*R* = 0.14 with *p* = 0.46). Fifteen subjects (35%) reported that they “don't know” if they snore or not. Of these subjects, we found that fourteen (93%) objectively snored (OSI>40 dB).

## Discussion

Our study proposes a robust snore detection system based on audio signals recorded using a non-contact microphone technology with overall accuracy rate of 98.2%. The novelty of our proposed system is its automatic detection of a variety of snore events from a whole-night audio recording. Our approach to snore detection includes comprehensive sets of features that were selected using the feature selection algorithm.

### Subjects

Sixty-seven typical subjects referred to PSG evaluation of sleep disorders were included in this study. No significant differences in subjects' demographic characteristics, age, BMI, AHI, and ESS were found between groups (Table 2). We included subjects referred to in-laboratory PSG evaluation including a wide range of age, BMI, and AHI distribution, and both genders. Further studies are needed to confirm our findings in the general population and in at-home settings.

In this study, we included 107,392 snore events, acoustically ranging from very soft to very loud (20–80 dB). The majority of these snoring events (97.5%) occurred predominantly during inspiration with minimal snoring energy during expiration (Fig. 4), similar to prior studies that concluded that expiratory snoring is relatively rare [31], [47]. Most studies [1], [4], [28], [31]–[33] have defined snores as acoustic events with sound intensity exceeding a certain amplitude value. Other studies have defined them as acoustic events that contain an oscillatory component [30] and even as any sound perceived as such by the observer holding the microphone [29]. Moreover, some studies have explored only selected snores (usually loud events) and did not analyze the whole sleep [48]. Since there is no unified approach to exactly define a snore, and it is more “in the ear of the beholder” [4], we included every noisy inhalation sound made during sleep that was >20 dB, i.e., the minimum sensitivity of our recording device.

We noted that the ratio of noise events to snore events was higher in the validation group compared to the design group (Table 2). This finding may be explained by the fact that the validation dataset contains a longer recording time (∼30 minutes), predominantly prior to lights off. Since total sleep time (and sleep efficiency) was statistically similar between the design and validation datasets (design: 343±48 minutes, validation: 349±42.0 minutes, *p* = 0.594), this extra recording time contained more noise events, which were mainly generated by patients and technicians prior to lights off. Nevertheless, the vast majority of the additional noise events were successfully detected by our system (>98%). This strengthens our hypothesis that snore events can be successfully detected even in a noisier environment since the training process (in the design phase) included a large variety of noise and snore events.

### Snore recording

We used a non-contact microphone to record snoring. This approach was challenging since it was essential to improve SNR in order to expose the acoustic signals that were of interest. To achieve this, we used an adaptive spectral subtraction technique that subtracted the estimated adjacent background noise. The spectral subtraction technique improved signal SNR with a minimal distortion effect on sound intensity. Our findings are supported by Karunajeew et al. [25], who explored the effect of signal enhancement using spectral subtraction prior to a snore detection system on a sample of 12 patients (8 and 4 were included in the design and validation studies, respectively) undergoing PSG. Karunajeew et al. showed that the detection rate could be improved from 90.7% to 96.7%. This technique of signal enhancement is very acceptable in the speech enhancement field, but has not been fully explored in nocturnal sound analysis. Recently, this process was found to be beneficial in cases where the speech signal was contaminated with loud background noise. Lee et al. [48] removed estimated background noise from an entire audio signal using a fixed filter. Their estimation was based on the spectrum from the initial ten minutes of the recording (empty room). This approach was better than most fixed noise reduction techniques (such as linear time invariant filter), but it did not follow the background noise properly through the night. To overcome the SNR challenge, some studies used a contact microphone, e.g., the tracheal microphone [19], [26], [27], [49]; however, data were easily affected by a variety of noises such as cardiac and respiratory sounds and movements. Other studies used a microphone embedded in a face mask [50]. In our case, we developed an adaptive and sensitive event detection algorithm based on energy measurement. This algorithm has proven to be significant when whole-night snoring event detection is necessary. A previous study [48] using an adaptive energy-related threshold supports our findings.

### Acoustic features

In this study, we established and explored a large and comprehensive set of features that are the most relevant for snore detection using a feature selection technique. In order to fully explore this variety of features, we have used concepts from speech and audio signal processing areas [22], [43] and implemented features from time and spectral domains (Table 1 and Table S1 in File S1). In addition, for the purposes of this study, we developed a novel and unique set of features that are specifically suitable for measuring breathing patterns (snores) during sleep. It is worth noting that the most influential feature set was the novel periodicity set (Table 1, Fig.5), a set of features found to be highly discriminative in classifying snore and non-snore events. The best selected individual feature (*E_P_*) belongs to this periodicity set and yielded an accuracy rate of 83.6% when tested alone on the validation dataset. However, detection of snoring events solely by this feature will not provide the desired performance. Therefore, complementary information from both time and spectral domains contributes considerably to improve our system's performance. Other studies have explored relatively few features based solely on spectra distribution [24], [26] or on an event's energy and duration [48]. Karunajeewa et al. [25] did use the two domains' (time and spectra) energy signals with a combination of zero crossing rate, autocorrelation at 1 ms lag, and the first LPC coefficient; their results show the potential of information taken from both domains based on a small database. Some studies have tried to reduce the dimensionality of feature space using principle component analysis (PCA) [24], [26] or Fisher's linear discriminant (FLD) [51]. Projecting the features from high dimensionality into a low dimension feature space indeed reduce the complexity of threshold decision making but ignore complicated and tangled patterns (of events). Hence, these kinds of approaches can be problematic since some of the inherent discrimination information between the classes can disappear. One of our study innovations was applying a feature selection algorithm (Table S1 in File S1) in order to evaluate a large set of features. This feature selection approach aimed to retain the discrimination ability between classes while significantly reducing the number of features.

For the design phase, we used the AdaBoost classifier. In preliminary studies [52], we also explored the effect of different classifiers such as a Gaussian mixtures model (GMM). The AdaBoost classifier was chosen because it was found to produce the best results. According to Fig. 7, the difference between the resubstitution and the ten-fold techniques in the optimal feature dimension (*d* = 34) was not greater than 1%, implying that there is no over-fitting even in a hyper-dimensional feature subset.

Body posture during sleep may affect the acoustic characteristics of snores, such as snoring intensity. Since body posture can change several times during sleep, we extracted a variety of features, exploring different normalization processes, and then applied the feature selection approach in order to objectively find the most robust features. Thirty-four features were selected (Table S2 in File S1). Among them, only 3 were energy-related. These features are not affected by the absolute event energy *per se*; i.e., two of the energy features (Tc_4_ and Tc_6_) are versions of a normalized energy, and the third feature (Tc_8_) is related to the event energy shape (skewness). Further study should investigate the effect of sleep position on snore detection.

### System performance

Comparing our snore detection performance with other studies, our results (accuracy>98%) are superior, especially when they are statistically proven based on a large dataset containing a variety of sleep disorder breathing (SDB) severity and recorded using an ambient microphone. Azarbarzin et al. [26] achieved an accuracy rate of 93.1% when using an ambient microphone. Karunajeewa et al. [25] published a high accuracy rate of 96.8% when testing four subjects. Cavusoglu et al. [24] produced an accuracy rate of 90.2% when recording using an ambient microphone placed 15 cm from the patient's head. Duckitt et al. [23] achieved 82–87% when trained and tested on a total of 6 snorers. We confirmed the robustness of our detector by using an additional audio recording device (handy *Olympus LS-5*) placed on the dresser beside the patient's head in the laboratory. Similar accuracy rates of 98.4% and 97.8% were found for the *Edirol R-4 Pro* (*n* = 30) and *Olympus LS-5* (*n* = 12, *p* = 0.14), respectively. Further studies are required to explore the usefulness of this snore detection in at-home settings.

Although expiratory sounds are relatively rare (see above), they were not excluded from our study. The snore/non-snore detection results include all the automatically detected sound events, including expiratory sounds. By analyzing the false-detected events (<2%), we noticed that the majority of the misclassified events were between inspiratory and expiratory snores. Further studies are required to explore these types of expiratory sounds.

### Objective measure of snoring

This study provides a novel and useful tool to objectively quantify a whole night's snoring events. Early work has concluded that to a large extent snoring is a subjective evaluation [4] when in fact the problem lies with the bed partner being disturbed by essentially normal nocturnal breathing noise [1]. This study shows a poor correlation between self-reported snore intensity and the objective measurement of snoring. This could be related either to the fact that subjects “do not know” or to biased bed partner reporting. Fig. 10 demonstrates a clear whole-night objective visualization tool for a snoring pattern that may aid clinicians in their clinical evaluations of snoring by presenting the subject's snoring index (snoring events per hour of sleep) and intensity (in dB). An innovative parameter for objectively scoring snore intensity was developed in this study. The OSI score was calculated using the detected snores, for the first time allowing very accurate and objective scores regarding the controversial self-reporting questionnaires. This simple tool can provide objective numeric and graphic reports of the automatically detected snoring events (Fig. 11, open bars). Another application for snore detection is to diagnose SDB syndromes [20], [27], [30], [48], the effectiveness of palatal surgeries regarding snores, OSA [18], and even exploration of breathing patterns during sleep time [33].

## Summary

One of the main goals of medicine today is to improve early diagnosis and treatment. Clearly, incidence of snoring is very frequent, and it is a common symptom of sleep-disordered breathing and other disorders of the upper airways [4]. The “flood” of subjects presenting with snoring symptoms is a major challenge to decision makers and is governed by prevalence and level of awareness of snoring morbidity [14]. Here, we have proposed a snore detection system that can provide an objective quantitative measure for whole-night snore patterns. Further studies are needed both to reinforce our findings by recruiting subjects from primary care clinics and by validating this snore analysis method as a potential screening tool in an at-home environment.

## Supporting Information

File S1 Online Methods Supporting Information.
(PDF)
