# Supplementary material for: Automatic Detection of Whole Night Snoring Events Using Non-Contact Microphone
Source: PLoS One. 2013 Dec 31;8(12):e84139. doi: 10.1371/journal.pone.0084139 (PMC3877189; doi:10.1371/journal.pone.0084139)
Supplement: File S1 — Online Methods Supporting Information. (PDF) [file pone.0084139.s001.pdf]

On line METHODS supplement

Automatic Detection of Whole Night Snoring Events Using Non-Contact Microphone

Eliran Dafna, Ariel Tarasiuk, Yaniv Zigel

ONLINE METHODS SUPPLEMENT

Study Protocol

A system-design study was performed in which a system for automatic detection of whole night snoring events was developed (Fig. 1 in the main text). The preprocessing step included noise reduction based on spectral subtraction in order to enhance the audio signal and improve the robustness of the system. Distinguishing snore and non-snore events was performed using ad hoc acoustic features that were developed and collected for this purpose. Thirty-four features were chosen using a feature selection technique that was designed to extract the most discriminative features, while removing those that were irrelevant. The chosen features were fed into an AdaBoost Classifier to produce a single snore-likelihood score using an individual score threshold, and the accuracy rate of performance was improved.

Presented here is supplemental technical information regarding the snore detection process.

Preprocessing (Adaptive noise suppression)

Each audio signal underwent an adaptive noise suppression (spectral subtraction) process based on the Wiener-filter. This process relies on automatically tracking background noise segments in order to estimate their spectra and subtracts them from the audio signal [E1].

First, the audio signal was divided into 40 ms frames (50% overlapping). For each frame, frequency components were calculated using a discrete Fourier transform (DFT) technique. The suppression was done by suppressing each frequency's magnitude, while leaving its phase unchanged according to the noise spectral template.

This template was initially estimated from the lowest energy frame of the first 10 sec of the audio signal and was updated during the adaptive noise suppression process.

Updating the template occurs when one of two conditions is met: 1) the current processed frame is assumed to be a background noise candidate, or 2) no background noise candidates were found in the

previous 10 sec.

Adaptation was calculated using weighted averaging of 10% from the new noise candidate and 90% from the old noise spectral template. When no noise candidate is found, it is forced to find a noise candidate (the minimal frame energy) in the last 10 seconds.

The frequency suppression factor $G(k)$ was determined using an estimation of *a priori* SNR of the processed frame at time index t and limited to the range [0, -25dB] in order to prevent a major distortion when low SNR was present. See Eq. (S1)

$$G^t(k) = \max\left(\frac{SNR^t_{prio}(k)}{SNR^t_{prio}(k)+1}, -25dB\right) \quad \text{(S1)}$$

where $G(k)$ is the suppression factor corresponding to the kth frequency component, t represents the frame index at time t, $SNR_{prio}(k)$ is the *a priori SNR* of the kth frequency component, and N_k represents the template components of the noise frequencies.

Defining local *a posteriori* and *a priori* SNRs by Eq. (S2) and Eq. (S3), respectively:

$$SNR^t_{post}(k) \triangleq \frac{\left|X^t_k\right|^2}{\left|N_k\right|^2} \quad \text{(S2)}$$

$$SNR^t_{prio}(k) \triangleq \frac{\left|X^t_k - N_k\right|^2}{\left|N_k\right|^2} = SNR^t_{post}(k) - 1. \quad \text{(S3)}$$

The right side of equation S3 is achieved due to the uncorrelated assumption between desired signal and noise. In this study, we modified and smoothed the *a priori* SNR using a weighted parameter α with the previous estimated *a priori* SNR, as presented in Eq. (S4):

$$SNR^t_{prio}(k) = (1-\alpha)\times\max\left(SNR^t_{prio}, 0\right) + \alpha\times\left|G^{t-1}(k)\right|^2 \times SNR^{t-1}_{prio}(k) \quad \text{(S4)}$$

where the max argument was designed to constrict the prior SNR to be positive, and α was chosen arbitrarily to be equal to 0.99 in order to refine the changes. After applying the suppression to the frames' magnitude frequencies ($\tilde{X}^t_k = X^t_k \times G^t(k)$), reconstruction of the signal was performed using the modified frequencies' magnitude and the corresponding unchanged phases.

Event detection and segmentation

In this study, we developed an event detection module for the purpose of detecting any acoustic/energetic event that could be a snore candidate according to its energy threshold and event duration rule. Moreover, the module was designed to achieve high sensitivity for detecting any energetic event including very low intensity snores.

Figure S1 presents the block diagram of the event detector module.

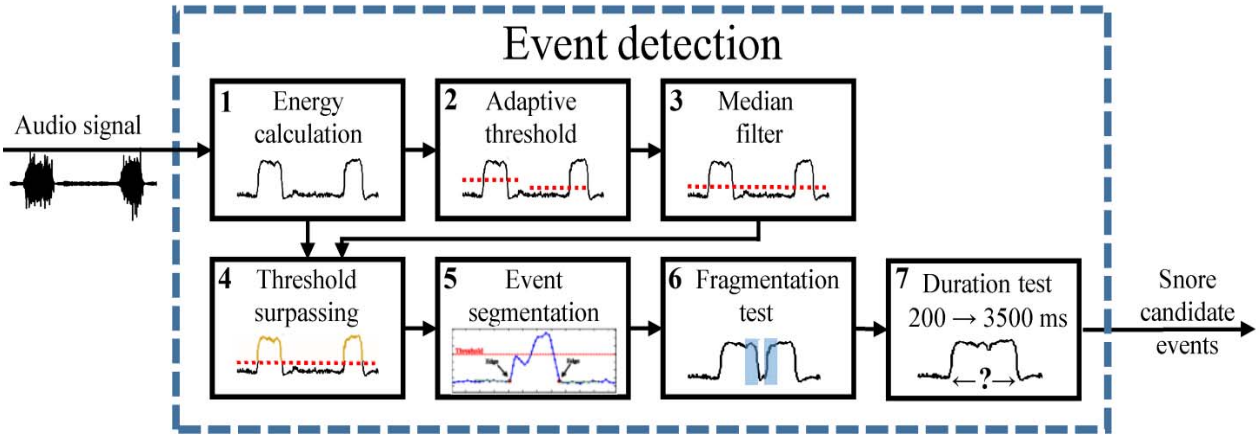

Figure S1 – Block diagram of the event detection module.

Block 1: The full-night audio signal was divided into one-minute sections. For every section, an energy vector was calculated using energy frames (frame size: 60 ms and 75% overlap).

Block 2: A section-related energy threshold (adaptive threshold) e_{th} was calculated using a histogram of the energy vector $hist_{energy}(e)$. Since the prevalence of stationary background noise frames was greater relative to the audio events (snore/non-snore), the energy value that is related to the peak of the histogram e_{max} was located in the low energy scale:

$$e_{max} = \arg\max_{e} \left\{ hist_{energy}(e) \right\}. \quad \text{(S5)}$$

The energy threshold ($e_{th} > e_{max}$) was set to the energy value corresponding to one-tenth of the peak amplitude:

$$hist_{energy}(e_{th}) = 0.1 \times hist_{energy}(e_{max}) . \quad \text{(S6)}$$

See the example in Fig. S2B.

Block 3: A five-order median filter was applied to the whole-night threshold values (vector) to smooth outliers.

Block 4: Applying the thresholds to the corresponding energy signal sections, suspected events are emerging, leading to starting and ending points of each suspected event.

Block 5: *Event segmentation* – In order to find the exact event boundaries (edges), the time edges of each audio event were calculated using an estimated slope technique. An illustration of the segmentation process is shown in Fig. 2 (in the main text). This process included the estimation of a slope from ten consecutive energy frames (150 ms window) – a linear regression fitting line was calculated from the consecutive energy frames in order to estimate its slope. This process was repeated and progressed outside the event boundaries one frame at a time as long as the slope did not change its sign.

Block 6: *Fragmentation test* – In case the detected audio events are too close to each other (< 200 ms), they were suspected to be one fragmented event (such as split snores). This fragmentation test involved a spectral similarity measure for the 100 ms adjacent windows of these events (the ending part from the first event and the initiating part from the following one). In case of similarity, the events were merged to form a single event.

Block 7: *Event duration test* – Only 200 ms to 3500 ms events were used in this study since we noticed that the duration of >99% of the manually labeled events fell in this range. Fig. 3 (in the main text) shows snore statistics based on the manual labeling of snoring events.

Figure S2 presents key steps in the event detection process. Panel A represents the energy signal associated with a one-minute segment. Panel B represents the corresponding histogram of the energy

signal. Panel C represents the surpassed energy signal using the filtered threshold. Note that this panel is prior to event segmentation, fragmentation, and duration tests. Panel D represents a section within Panel C after all the tests and rules were applied, i.e., the final outcome.

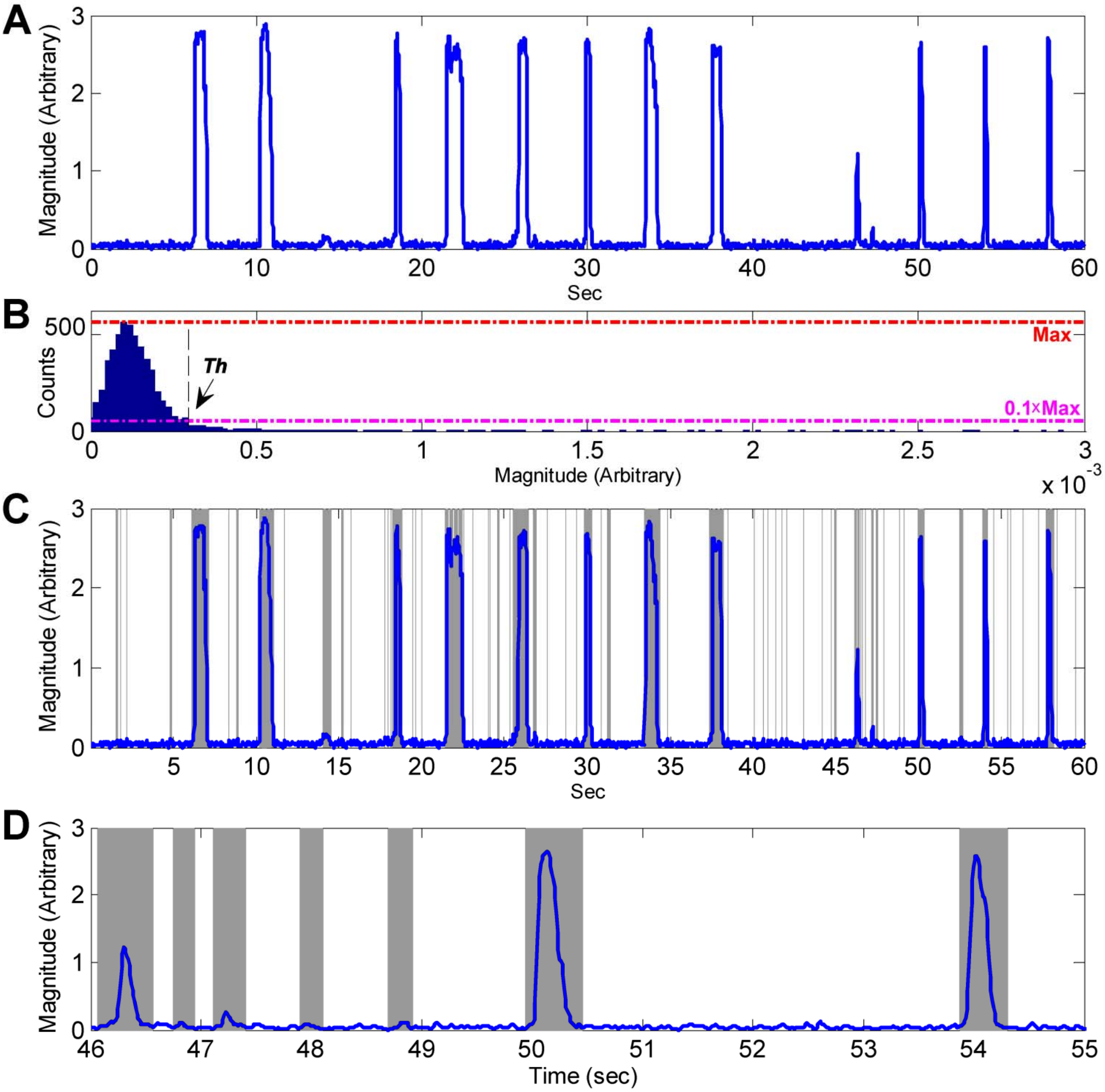

Figure S2 – Example of key steps in the event detection process. A) Energy calculation of a one-minute segment. B) The corresponding energy value histogram. The threshold was calculated according to Eq. (S6). C) The surpassed signal according to the filtered threshold. The grey background represents the event boundaries. Note that at this stage there was very high false alarm detection. D) The final detection output with zoom in at the corresponding C panel. Note that the remaining events surpassed every test and rule.

In order to validate our event detection module, we arbitrarily analyzed a total of four hours and ten minutes of recordings taken from 25 subjects (10 minutes from the middle of the recording for each subject from the design set).

The analysis process included manually marking each detectable audio event. The events were detected by listening and visually observing the energy and spectrogram of each suspected signal section. In total, we manually marked 3429 events, 137±66 per subject.

We automatically detected 592±171 snores per subject, resulting in *TP*=100% (positive detection) of manually detected events and with positive predictive value *(PPV)* of 23.1%, (×4 more false alarms).

Agreement between scorers

Manual labeling of audio events was an essential step for designing and evaluating the snore detection algorithm. Therefore, an *ad hoc* graphical user interface (GUI) for manual event classification based on visual and acoustic perception of the event itself and its surrounding context was designed, and research assistants (scorers) were rigorously and uniformly trained (see main text). We used Cohen's kappa coefficient [E2] to estimate the agreement between the three scorers. The following confusion matrices (CM) between each pair of the three scorers were obtained:

$$CM_{1,2} = \begin{pmatrix} 0.9955 & 0.0045 \\ 0.0173 & 0.9827 \end{pmatrix},\quad CM_{1,3} = \begin{pmatrix} 0.9986 & 0.0014 \\ 0.0156 & 0.9844 \end{pmatrix},\quad CM_{2,3} = \begin{pmatrix} 0.9965 & 0.0035 \\ 0.0209 & 0.9791 \end{pmatrix}.$$

Very high agreement κ_{12}=97.4%, κ_{13}=97.9%, and κ_{23}=97.0% corresponding to that between scorers 1 and 2, scorers 1 and 3, and scorers 2 and 3, respectively, was achieved.

Feature extraction

Table S1 summarizes all the features that were extracted based on time and spectra-related domain.

Table S1. **Features pool.**

Feature	Symbol*	Count	Feature	Symbol	Count
**I. Time related (Inter- & Intra-events)**	T	**25**	**II. Spectra-related (Intra-events)**	S	**102**
**a) Periodicity features (Inter-events)**	$Ta_{\bullet}$ *	**10**	**a) Spectra models**	$Sa_{\bullet}$	**68**
1. Rhythm period (±6 sec)	Ta_1	1	1. 20-MFCC (model coefficients)	$Sa_{1\#1:20}$	20
2. Rhythm period (±12 sec)	Ta_2 τ_p	1	2. 20-LPC (model coefficients)	$Sa_{2\#1:20}$	20
3. Rhythm intensity (±6 sec)	Ta_3	1	3. 8-subband-frequency distribution	$Sa_{3\#1:8}$	8
4. Rhythm intensity (±12 sec)	Ta_4 R_I	1	4. MFCC (4 moments of coefficients)	$Sa_{4\#1:4}$	4
5. Relative energy prior to event	Ta_5 E_P	1	5. LPC (4 moments of coefficients)	$Sa_{5\#1:4}$	4
6. 10 sec after (event's normalized area)	Ta_6	1	6. LP residuals (4 moments of residuals)	$Sa_{6\#1:4}$	4
7. 10 sec area differences	Ta_7	1	7. DFT (4 moments of freq. distribution)	$Sa_{7\#7:4}$	4
8. 10 sec area division	Ta_8	1	8. DFT (4 moments of amp. distribution)	$Sa_{8\#1:4}$	4
9. 10 sec before & after period ratio	Ta_9	1	**b) Bio-characteristic frequency**	$Sb_{\bullet}$	**10**
10. 10 sec before & after correlation	Ta_{10}	1	1. 3-first formants (frequency)	$Sb_{1\#1:3}$	3
**b) Duration and sample scattering (Intra-events)**	$Tb_{\bullet}$	**4**	2. 3-first formants (magnitude)	$Sb_{2\#1:3}$	3
1. Duration	Tb_1	1	3. F3-F1 (difference in frequency)	Sb_3	1
2. Duration trimmed (95%)	Tb_2	1	4. Pitch	Sb_4	1
3. ZCR	Tb_3	1	5. Pitch intensity	Sb_5	1
4. Entropy	Tb_4	1	6. Pitch density	Sb_6	1
**c) Energy (Intra-events)**	$Tc_{\bullet}$	**11**	**c) Dynamic frequency**	$Sc_{\bullet}$	**24**
1. Intensity (dB)	Tc_1	1	1. Δ-MFCC	$Sc_{1\#1:20}$	20
2. SNR (dB)	Tc_2	1	2. Dynamic MFCC	Sc_2	1
3. Total area beneath energy envelop	Tc_3	1	3. Spectra flux	Sc_3	1
4. Normalized area beneath energy envelop	Tc_4	1	4. Centroid movement	Sc_4	1
5. Volume density rate (VDR)	Tc_5	1	5. MelCepstability	Sc_5	1
6. Ratio of areas before & after the peak	Tc_6	1			
7. Skewness of amplitudes	Tc_7	1			
8. Skewness of envelop formation	Tc_8	1			
9. Kurtosis of amplitudes	Tc_9	1			
10. Kurtosis of envelop formation	Tc_{10}	1			
11. Slope to 1st peak	Tc_{11}	1			

* The simplified symbols used in the paper body.

For convenience, the code name for each feature is composed from 3 symbols in its form: Xy_{num}, where "*X*" represents the domain {*T*=Time, *S*=Spectra}, "*y*" represents the domain subgroup {a,b,c}, and "$_{num}$" represents the index within subgroup y. For example, the code name for Pitch is Sb_4, and for the 10th MFCC coefficient, it is $Sa_{1\#10}$.

Time-domain set

Twenty-five features were included in this domain, categorized into three groups: a) Periodicity features, b) Duration and sample scattering, and c) Energy.

a) Periodicity features – Periodicity features are inter-event features based on a tested event's surrounding context. In fact, these features are derived from the energy signal corresponding to the interval that is being tested.

$\boldsymbol{Ta_1}$ and $\boldsymbol{Ta_2}$ are features that measure the period of the rhythm detected in a 12- and 24-second interval, respectively, when the tested event is in the middle. The period was calculated via auto-correlation over the energy signal associated with the tested interval (see Fig. 5 in the main text).

$\boldsymbol{Ta_3}$ and $\boldsymbol{Ta_4}$ are the corresponding intensities of the detected rhythm of Ta_1 and Ta_2. They were calculated using Eq. (3) in the main text.

$\boldsymbol{Ta_5}$ and $\boldsymbol{Ta_6}$ are the normalized area (relative to the tested event) preceding and following the event, respectively; basically these features measure the equally existing events prior to or after the event.

$\boldsymbol{Ta_7}$ and $\boldsymbol{Ta_8}$ are scores calculated over $\boldsymbol{Ta_5}$ and $\boldsymbol{Ta_6}$ simultaneously using subtraction or division of the two.

$\boldsymbol{Ta_9}$ is the ratio of periods calculated for the 10 sec prior to the event and 10 sec after the event; this feature is essential in detection of apneic snores.

$\boldsymbol{Ta_{10}}$ is a feature that measures the correlation coefficient between the 10 sec prior to the event and the 10 sec after the event.

b) Duration and sample scattering – This sub-group contains four features: two that measure the duration of the tested event and two that measure its sample scattering.

$\boldsymbol{Tb_1}$ is the duration (in sec) of the entire event (from start to end), while $\boldsymbol{Tb_2}$ is the shortest duration in seconds (within the event) that holds 95% of the event's total energy.

$\boldsymbol{Tb_3}$ is the zero crossing rate (ZCR) of the event samples. $\boldsymbol{Tb_4}$ is the entropy of the event samples measured as in Eq. (S7):

$$Tb_4 = -\sum_{i=1}^{M} P_i \log(P_i) \tag{S7}$$

where P_i is the probability of the amplitude to be quantified to bin i out of M=100 bins quants.

c) **Energy (Intra-events)** is the third sub-group within the time domain features. It contains features calculated regarding both event-based and frame-based energy features.

$\boldsymbol{Tc_1}$ and $\boldsymbol{Tc_2}$ are event-based energy features that measure the event's total intensity and the SNR of the event (both on the dB scale), respectively.

$\boldsymbol{Tc_3, \ldots, Tc_{11}}$ are frame-based energy features that measure parameters regarding the formation of the event's frames.

$\boldsymbol{Tc_3}$ is the total area beneath the event's energy and $\boldsymbol{Tc_4}$ is the normalized area beneath the event's energy, where the rectangle that contains the event is equal to 1.

$\boldsymbol{Tc_5}$ is the volume density rate (VDR). It is similar to the SNR score but calculated as [(max-min)/max] of the energetic frames.

$\boldsymbol{Tc_6}$ is the ratio between the areas located prior to and after the maximum peak location of the energetic frames.

$\boldsymbol{Tc_7}$ is the skewness (3rd moment) of the frame's magnitude distribution.

$\boldsymbol{Tc_8}$ is the skewness (3rd moment) of the frame's formation along time.

$\boldsymbol{Tc_9}$ and $\boldsymbol{Tc_{10}}$ are the same as $\boldsymbol{Tc_7}$ and $\boldsymbol{Tc_8}$ but calculate the kurtosis (4th moment).

$\boldsymbol{Tc_{11}}$ is the slope of the line connecting $E_{t=0}$ and $E_{t=peak}$. The slope is calculated as: $1/(t_{peak}-t_0)$.

Spectral-domain set

One-hundred-and-two features were included in this set, comprising three sub-groups: a) Spectra models, b) Bio-characteristic frequencies, and c) Dynamic frequencies.

a) **Spectra models** – This category includes coefficients of different spectra models and four moments of the distribution of their coefficients.

DFT_k denotes a 128-coefficient DFT of the event's signal.

Sa_1 is the 20th order MFCC model [E3] containing 20 coefficients as 20 individual features.

Sa_2 is the 20th order LPC model [E3]. It also contains 20 coefficients as 20 individual features.

Sa_3 represents 8 sub-bands of the event's DFT content,

$$S_i = \frac{\sum_{k=8i}^{8i+7} |DFT_k|}{\sum_{k=0}^{127} |DFT_k|} \tag{S8}$$

Sa_4 represents the four moments of the MFCC coefficient (**$Sa_{1\#1}$,..., $Sa_{1\#20}$**) distribution.

Sa_5 represents the four moments of the LPC coefficient (**$Sb_{1\#1}$,..., $Sb_{1\#20}$**) distribution.

Sa_6 represents the four moments of the LP residuals (the residual of the signal when applying the LPC model).

Sa_7 represents the four moments of the frequency distribution of the absolute DFT.

Sa_8 represents the four moments of magnitude of the absolute DFT distribution.

b) **Bio-characteristic frequencies** – This category includes features regarding models and parameterization of the human vocal tract presented in the event.

Sb_1 are three features that estimate the frequencies of the first three formants.

Sb_2 are three features that estimate the amplitude of the corresponding first three formants.

Sb_3 is the difference between the third and the first formants.

Sb_4 is the detected pitch within the event.

Sb_5 is the intensity of the detected pitch (i.e., the periodicity of the event).

Sb_6 is the pitch density [E4].

c) **Dynamic frequencies** – This category includes features that measure and quantify frequency changes through time.

Sc_1 are the delta-MFCC [E3]. They represent the first derivative over each coefficient through time (samples).

Sc_2 is the dynamic MFCC feature, which measures the MFCC's variance along time Eq.(S9):

$$Sc_2 = \frac{1}{20}\sum_{k=1}^{20} VAR\left[MFCC(\mathrm{k},\mathrm{n})\right]. \qquad \text{(S9)}$$

Sc3 is the spectra flux feature; it is measured as Eq. (S10):

$$Sc_3 = \frac{1}{8}\sum_{k=1}^{8}\frac{1}{N}\sum_{n=1}^{N-1}\left[\log\left(\left|DFT_{k,n+1}\right|\right) - \log\left(\left|DFT_{k,n}\right|\right)\right]^2 \qquad \text{(S10)}$$

where $DFT_{k,n}$ represents the DFT in frequency sub-band *k* and at the frame index *n*. *N* is the total number of frames of the event.

Sc_4 is the frequency's centroid transitions in event, which measures the distance traveled of the frequency center of mass calculated in a mel-scale as shown in Eq. (S11)

$$Sc_4 = \frac{1}{N}\sum_{n=1}^{N-1}\left|MelCenter_{n+1} - MelCenter_n\right| \qquad \text{(S11)}$$

where $MelCenter_n$ represents the center of mass of the frequencies in mel-scale at frame index *n*.

Sc5 is the MelCepstability feature [E4].

Feature selection

Table S2 presents the selected features that discriminate between snore and non-snore events according to the forward feature selection. Note that the contribution of the features to this separability is presented in descending priority order (left to right).

Table S2. Selected features.

#	Symbol	#	Symbol	#	Symbol	#	Symbol
1	Ta_5	10	$Sa_{1\#2}$	19	$Sa_{1\#6}$	28	$Sa_{2\#4}$
2	Ta_4	11	$Sa_{7\#1}$	20	Sc_2	29	$Sa_{4\#1}$
3	$Sb_{1\#1}$	12	$Sa_{1\#13}$	21	$Sa_{1\#16}$	30	$Sa_{3\#4}$
4	Tc_8	13	$Sa_{5\#2}$	22	Sc_3	31	Ta_1
5	Tc_4	14	Ta_2	23	Sb_6	32	Tc_6
6	$Sa_{4\#3}$	15	$Sa_{5\#1}$	24	$Sa_{3\#7}$	33	Sc_4
7	Sc_5	16	Ta_8	25	$Sa_{7\#4}$	34	$Sb_{2\#1}$
8	$Sa_{1\#5}$	17	$Sa_{2\#6}$	26	Sb_5		
9	Tb_4	18	Ta_9	27	$Sa_{1\#11}$		

References

E1. Scalart P (1996) Speech enhancement based on a priori signal to noise estimation. Conf Proc IEEE International Conference on Acoustics, Speech, and Signal Processing 2: 629-632.

E2. Cohen J (1960) A coefficient of agreement for nominal scales. Educational and psychological measurement 20: 37-46.

E3. Deller JR, Hansen JHL, Proakis JL (2000) Discrete-time processing of speech signals. New York: Institute of Electrical and Electronics Engineers Press.

E4. Ben-Israel N, Tarasiuk A, Zigel Y (2012) Obstructive apnea hypopnea index estimation by analysis of nocturnal snoring signals in adults. Sleep 35: 1299-1305C.
